# Femtosecond laser for cavity preparation in enamel and dentin: ablation efficiency related factors

**DOI:** 10.1038/srep20950

**Published:** 2016-02-11

**Authors:** H. Chen, H. Li, YC. Sun, Y. Wang, PJ. Lü

**Affiliations:** 1 Center of Digital Dentistry, Faculty of Prosthodontics, Peking University School and Hospital of Stomatology & National Engineering Laboratory for Digital and Material Technology of Stomatology & Research Center of Engineering and Technology for Digital Dentistry of Ministry of Health, 22 Zhongguancun Nandajie, Haidian District, Beijing, 100081, China

## Abstract

To study the effects of laser fluence (laser energy density), scanning line spacing and ablation depth on the efficiency of a femtosecond laser for three-dimensional ablation of enamel and dentin. A diode-pumped, thin-disk femtosecond laser (wavelength 1025 nm, pulse width 400 fs) was used for the ablation of enamel and dentin. The laser spot was guided in a series of overlapping parallel lines on enamel and dentin surfaces to form a three-dimensional cavity. The depth and volume of the ablated cavity was then measured under a 3D measurement microscope to determine the ablation efficiency. Different values of fluence, scanning line spacing and ablation depth were used to assess the effects of each variable on ablation efficiency. Ablation efficiencies for enamel and dentin were maximized at different laser fluences and number of scanning lines and decreased with increases in laser fluence or with increases in scanning line spacing beyond spot diameter or with increases in ablation depth. Laser fluence, scanning line spacing and ablation depth all significantly affected femtosecond laser ablation efficiency. Use of a reasonable control for each of these parameters will improve future clinical application.

Tooth preparation is a very basic and important operation in oral clinical work. However, the harsh noises produced by high-speed turbo drills commonly used in traditional tooth preparation tends to make the patients uncomfortable, and the pain caused by mechanical friction and heat requires the use of a local anesthetic^1^,^2^,^3^ Moreover, mechanical preparation of dentin produces a contamination layer^4^,^5^,^6^,^7^, ^8^,^9^,^10^ on its surface, affecting bond strength. The recent application of lasers for dentistry has shown promise in solving each of these problems^8^, ^9^, ^10^, ^7^, ^8^, ^9^, ^10^. An Er:YAG laser, which is a representative waterlaser, can effectively cut dental hard tissues^11^,^12^,^13^,^14^; however, its results do not always meet the strict accuracy requirements of tooth preparation for oral inlays, crowns and other restorations.

Femtosecond lasers are widely used in fields of micro-nanofabrication, with the notable advantage of having a high 3D machining accuracy. A femtosecond laser is an ultra-short pulsed laser whose pulse is less than one nanosecond (1 femtosecond = 10^-15^ seconds). Importantly, its laser energy can break out very quickly and produce power enough to ionize its target directly to plasma which then quickly dissipates, taking energy away and thus transferring little to no heat to surrounding tissues. With its ability for “cold” ablation and its machining accuracy achieving sub-micro and even nano levels^15^,^16^, the femtosecond laser appears to be a better choice than others for tooth preparation. Indeed, studies have described the favorable femtosecond laser ablation of dental hard tissue^5^,^17^,^18^. Other benefits are that the canal temperature can be controlled by cooling within a safe range, and the dentinal tubules remain open after cutting, so no smear layer forms^19^.

However, femtosecond lasers tend to be slow when cutting dental hard tissues. The ablation rate, AR, is defined as the volume of dental hard tissue cut by the laser per unit time (*AR = V/t*, mm^3^/s). To meet clinical requirements, the ablation rate must be maximized. Moreover, the ablation efficiency, AE, is defined as the volume of dental hard tissue cut by the laser per unit energy (*AE = V/E*, mm^3^/J). As power (*P*) equals *E/t*, then *AR = AE × P*, thus the ablation rate is determined solely by the ablation efficiency as long as the laser output power remains unchanged. Our previous studies^20^ showed that, when laser spot ablating in a line, laser energy density (fluence) has a very significant influence on ablation efficiency. As a clinically-prepared dental tissue cavity typically has a 3D form, to simulate clinical practice, this study built a 3D digital ablation platform where a rectangular cavity forms on enamel and dentin surfaces. In search of a suitable parameter to improve efficiency of femtosecond laser ablation, the relationships between laser fluence, scanning line spacing (distance between adjacent scanning lines), ablation depth and ablation efficiency were assessed.

## Materials and Methods

This study was approved by the bioethics committee of Peking University School and Hospital of Stomatology (PKUSSIRB-201522044). The procedures and risks involved with participation in this study were discussed with the volunteers, and written informed consent was obtained from each included participant. The methods were carried out in accordance with the approved guidelines.

### Material preparation

First molars removed in the clinic due to orthodontic needs were sterilized by soaking in formalin, then the roots were removed and the crowns retained. Using a diamond wire saw, the crowns were cut along the direction perpendicular to the longitudinal axis of the tooth into 1.5−mm thick samples consisting of an outer enamel ring of ~1−mm width and an interior dentin and pulp cavity structure. Following ultrasonic cleaning, the samples were kept in physiological saline.

### Platform construction

A diode-pumped, thin-disk femtosecond laser (JenLas^®^ D2.fs, Jena, Germany) with a wavelength of 1025 nm and pulse width of 400 fs was used in this study. The repeat frequency rate of 30~200 kHz and output power of 0 ~ 4 W can be adjusted by the matching software. The focus spot of diameter 24 μm, as measured by CCD (charge-coupled device), was guided by a vibrating mirror system (GO2-YAG-12-22-D, Beijing Golden Orange Technology Co., Ltd., China) and its control software (EasyCad V1, Beijing Golden Orange Technology Co., Ltd., China). The ablation volume was calculated using a 3D measurement laser microscope (Keyence VK-X100/X200, Keyence, Japan).

**Figure 1:**
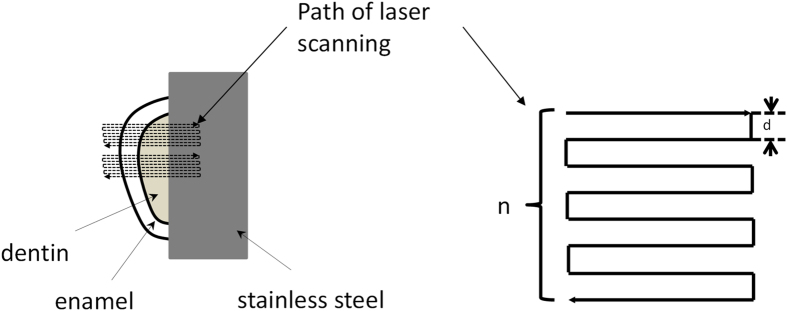
Schematic of laser ablation path. d: scanning line spacing; n: number of scanning lines in one layer.

As shown in Fig. 1, an experimental platform for laser ablation of the sample was constructed *in vitro*. After fixing teeth samples on the platform, about 2/3 of the dental area was covered by a stainless steel blade. The laser beam with vertical incidence and focused on the dental surface and was guided using a rectangular wave scanning motion. The scanning line passed across the boundary line of enamel and dentin resulting in a rectangular cavity with a width of ~0.5 mm.

### Settings to determine the effect of laser fluence on ablation efficiency

In this article, laser fluence (*F*) was calculated from the following formula^21^


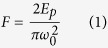


where *Ep* is the energy of a single laser pulse which equals laser power (*P*) divided by laser repetition frequency (*R*), and 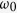 represents waist diameter of laser focus spot.

Scanning speed was set at 720 mm/s, scanning line spacing at 24 μm, diameter of laser focus spot measured to be 24 μm, repetition frequency of laser pulse 30KHz, and the number of scanning lines in one layer was 20. Laser power was set at 230, 460, 690, 920 and 1150 mW; the corresponding laser fluence was 1.56, 3.13, 4.69, 6.25 and 7.81 J/cm^2^; and the corresponding number of scanning layers was 800, 800, 400, 200 and 200. The number of scanning layers was reduced at higher laser fluences to avoid creating too large of an ablation depth and to ensure that the ablation surface is located near the effective range of the laser focal point.

### Settings to determine the effect of scanning line spacing on ablation efficiency

Scanning speed was set at 720 mm/s, laser power at 690 mW, and the corresponding laser fluence at 4.69 J/cm^2^. Scanning line spacing was then set at 6, 12, 25, 30, 40 and 50 μm; and the corresponding number of scanning lines in one layer was 83, 42, 20, 17, 13 and 10 to maintain a cavity width of ~500 μm. To keep the ablation time of each group as close as possible, the corresponding number of scanning layers was 100, 200, 400, 500, 600 and 800.

### Settings to determine the effect of ablation depth on ablation efficiency

Scanning speed was set at 720 mm/s, laser power at 690 mW, and the corresponding laser fluence at 4.69 J/cm^2^. Scanning line spacing was then set at 12 μm; the corresponding number of scanning lines in one layer was 42; and the corresponding number of scanning layers was 5, 10, 25, 50, 75, 100, 200, 300 and 400.

### Measurement and calculation of ablation efficiency

To calculate ablation efficiency, the time (*t*) spent by the laser cutting into the cavity, whose length is *l*, was first calculated as:


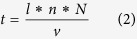


where *n* is the number of scanning lines within one layer, *N* is the number of ablation layers, and *v* is the laser scanning speed. Therefore, ablation rate can be calculated:


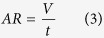


where *V* is the volume of the cavity. Ablation efficiency (*AE*) can be calculated as:


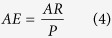


where *P* is the average power of the laser. Combining formulas (2), (3) and (4) produces the following formula of ablation efficiency:


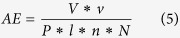


**Figure 2:**
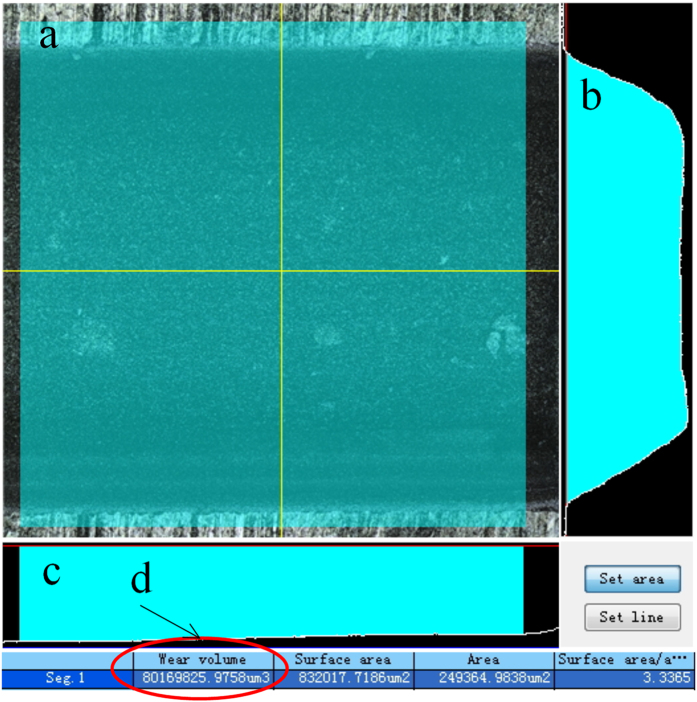
Measurement of cavity volume using a 3D laser scanning microscope. (**a**) selection of the measurement area (green area); (**b**) cross-sectional profile of cavity (yellow vertical line in part (**a**));(**c**) cross-sectional profile of cavity (yellow horizontal line in part (**a**));(**d**) automatic calculation by software of cavity volume, comprised of selected area, side walls and bottom wall.

The three-dimensional morphology of the cavity was obtained using a 3D laser scanning microscope. Selecting a *l* = 500 μm region in the ablated field, the supporting software automatically calculated the enclosed volume comprised of the selected region, the side walls and the bottom wall (Fig. 2). Ablation efficiency was then calculated using formula (5). Every cavity in each sample was measured 4 times for repeat.

## Results

### Effect of laser fluence on ablation efficiency

**Figure 3:**
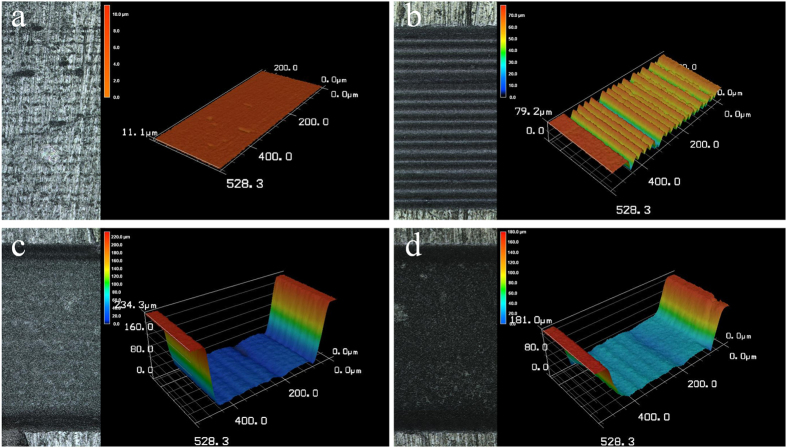
3D graphical representation of surfaces ablated at different laser fluences. At a laser fluence of 1.56 J/cm^2^, scanning line spacing of 24 μm and number of scanning layers of 800: (**a**) enamel and (**b**) dentin; At a laser fluence of 6.25 J/cm^2^, scanning line spacing of 24 μm and number of scanning layers of 200: (**c**) dentin and (**d**) enamel.

**Figure 4:**
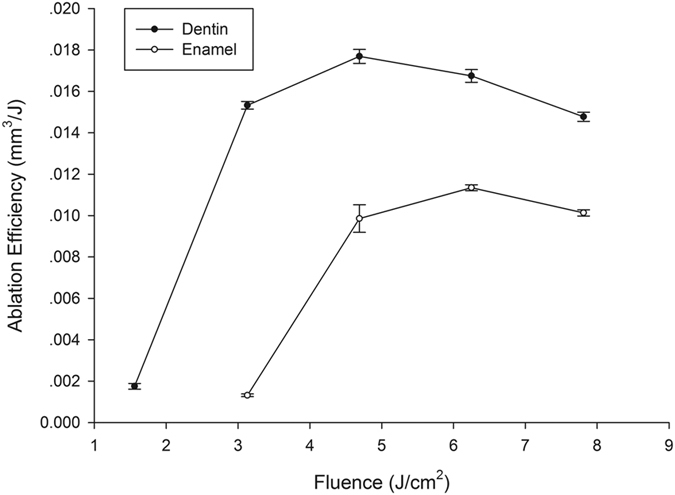
Relationship between laser fluence and ablation efficiency for enamel and dentin.

At a laser fluence of 1.56 J/cm^2^, enamel surface ablation was not observed; however, parallel lines of a shallow depth were observed on the dentin surface (Fig. 3a,b). Under higher laser fluence, the cavity bottom became smoother, and the ablation depth increased. Notably, the cavity showed a gully morphology and was wider near the top and narrower near the bottom (Fig. 3c,d). Ablation depths of enamel and dentin were observed at different combinations of laser fluence and number of layers scanned (Table 1), all the depth were controlled below 0.5mm to keep the ablating surface within laser focus. The relationship between laser fluence and ablation efficiency on enamel and dentin is shown in Fig. 4. Generally, ablation efficiency increased with an increase of laser fluence up to a certain threshold and then began to decrease. The maximum ablation efficiency for enamel was 0.0114 ± 0.0001 mm^3^/J at a 6.25 J/cm^2^ laser fluence and 200 scanning layers, while the highest ablation efficiency for dentin was 0.0177 ± 0.0003 mm^3^/J at a 4.69 J/cm^2^ laser fluence and 400 scanning layers. Past these points, increases of laser fluence resulted in gradual decreases of the ablation efficiencies, which is a finding that is in agreement with our previous experiments^20^

### Effect of scanning line spacing on ablation efficiency

**Figure 5:**
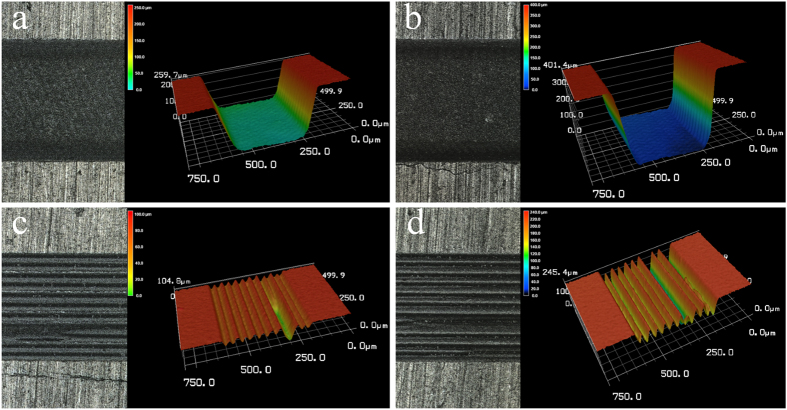
3D graphical representation of surfaces ablated at different scanning line spacings. At a laser fluence of 4.69 J/cm^2^, scanning line spacing of 12 μm and number of scanning layers of 200: (**a**) enamel and (**b**) dentin; At a laser fluence of 4.69 J/cm^2^, scanning line spacing of 40 μm and number of scanning layers of 600: (**c**) enamel and (**d**) dentin.

**Figure 6:**
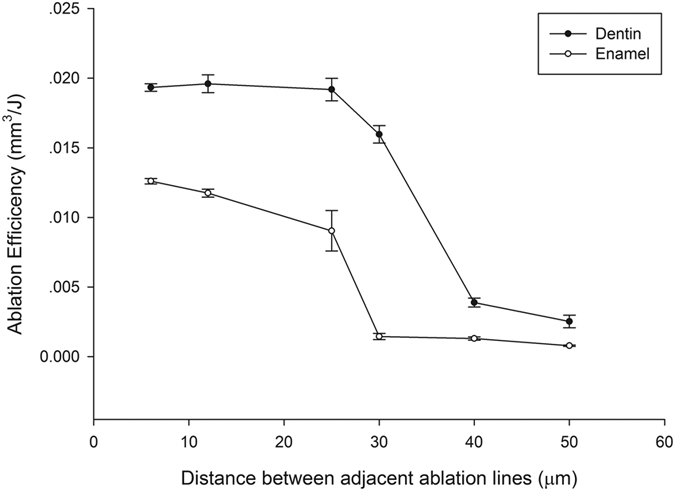
Relationship between scanning line spacing and ablation efficiency for enamel and dentin

For both enamel and dentin, at scanning line spacing less than the laser spot diameter (24 μm), the cavity bottom was relatively flat and smooth (Fig. 5a,b); when greater than the spot diameter, however, the cavity bottom was uneven and formed a periodic peak repeat, or a valley morphology, signifying a dramatic decrease in ablation efficiency (Fig. 5c,d). The relationship between scanning line spacing and ablation efficiency at a laser fluence of 4.69 J/cm^2^ is shown in Fig. 6.

### Effect of ablation depth on ablation efficiency

**Figure 7:**
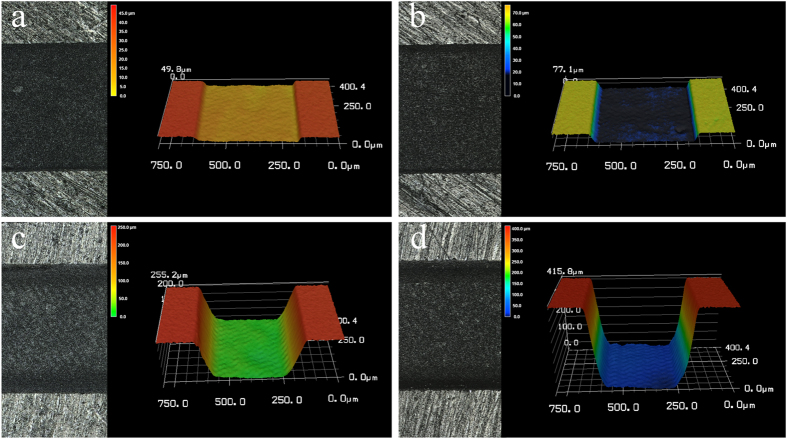
Ablation surfaces and their 3D graph in different number of ablation layers. At a laser fluence of 4.69 J/cm^2^, scanning line spacing of 12 μm and number of scanning layers of 25: (**a**) enamel and (**b**) dentin; At a laser fluence of 4.69 J/cm^2^, scanning line spacing of 12 μm and number of scanning layers of 200: (**c**) enamel and (**d**) dentin.

**Figure 8:**
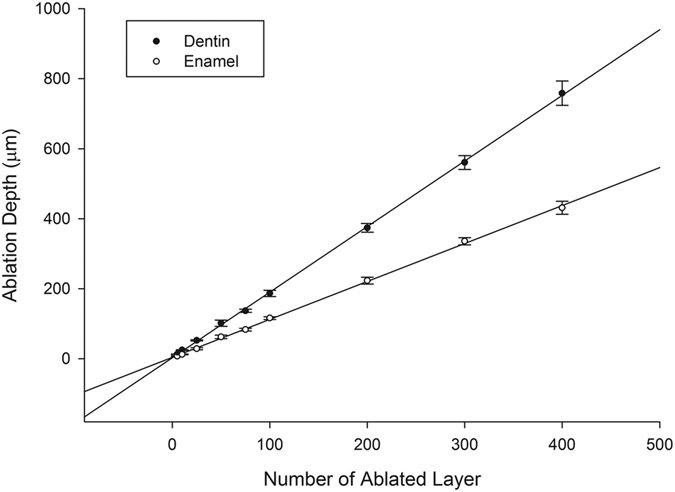
Relationship between number of ablated layers and ablation depth for enamel and dentin, a linear relationship was revealed (dentin: R^2^ = 0.997; enamel: R^2^ = 0.997).

**Figure 9:**
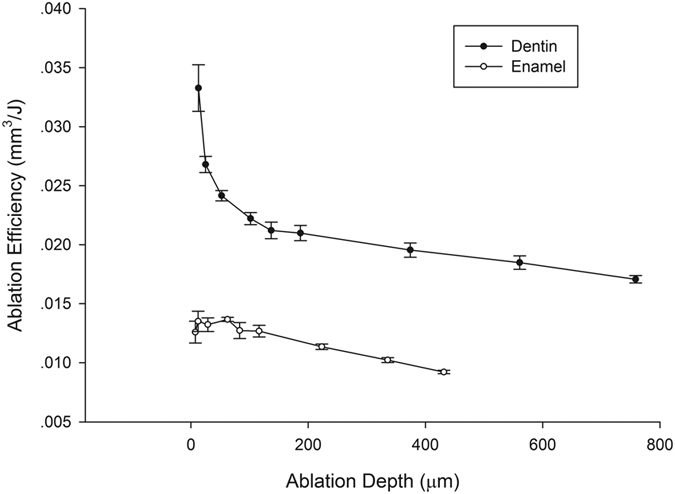
Relationship between ablated depth and ablation efficiency for enamel and dentin.

Increasing of the number of scanning layers also increased the depth of the ablation cavity (Fig. 7), and a linear relationship between them was revealed (Fig. 8). The relationship between ablation depth and ablation efficiency is shown in Fig. 9. For enamel and dentin, ablation efficiency decreased with an increase of ablation depth (or ablated layers); however, ablation efficiency for dentin dropped sharply at ablation depths of 0~20μm, while the drop rate decreased with increasing depth.

## Discussion

Previous studies have described the close relationship between femtosecond laser fluence (*F*) and ablation depth (*L*) of a single pulse^22^


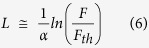


Where *α* is the effective absorption coefficient of the material for laser light and *F*_*th*_ is the ablation threshold value of the material (J/cm^2^). From this formula, one can see that as the laser fluence increases, both the ablation depth and volume of a single laser pulse (*V*_*p*_) increase. However, the increase of laser fluence depends on a proportional increase of laser pulse energy (*E*_*p*_) (see equation (1))^21^

According to the definition in this study, *AE = V*_*p*_*/E*_*p*_. When *F* increases, *V*_*p*_ and *E*_*p*_ also increase, which makes any change in *AE* more complicated. The results show that the ablation efficiencies for enamel and dentin increase and then fall with increasing laser fluence, which is consistent with the experimental results of our previous study^20^. One possible reason for this non-linear change is that, when laser fluence is small, *V*_*p*_ increases faster than *E*_*p*_ when *F* increases, so *AE* improves. However, when *F* is larger, the rate of increase of *V*_*p*_ is less than that of *E*_*p*_ when *F* also increases, thereby reducing *AE*. Further theoretical study of the quantitative relationship between laser fluence and ablation efficiency is needed.

The influence of scanning line spacing on ablation efficiency in this study was quite unexpected. Owing to that, the laser fluence (4.69 J/cm^2^) used for study of scanning line spacing was far greater than the ablation threshold of enamel (0.6 ~ 2.2 J/cm^2^) or dentin (0.3 ~ 1.4 J/cm^2^)^17^,^18^,^23^,^24^,^25^,^26^,^27^, and the enamel and dentin surfaces were effectively cut within the light spot diameter range. When scanning line spacing was less than the effective ablation diameter, two adjacent ablation lines overlapped, and the material was sufficiently removed. No residual enamel or dentin was present between scanning lines, and a rectangular cavity with a relatively flat bottom surface was produced, so ablation efficiency was high. When the scanning line spacing was greater than the effective cutting diameter, enamel and dentin between adjacent ablation lines was not removed, so a peak and valley pattern emerged. This uneven surface morphology may be the result of any of four mechanisms: energy coupling (reduced laser intensity due to increased surface area), heat conduction (decreased surface temperature at the tip due to three-dimensional heat diffusion), plasma shielding, or impediment of material expulsion^28^

Femtosecond laser energy follows a Gaussian distribution; that is, energy density decreases from the center of the laser focal point outward. So when a single pulse focuses on the surface of a dental hard tissue, the cutting depth of the central region is greater than surrounding areas, forming a certain degree of taper. Use of multiple pulse scanning lines forms a recessed region with a particular axial wall angle, while rectangular wave scanning forms a trapezoidal cross-section. Reasonable control of the taper of the ablated region is very important for correct insertion and retention of the dental prosthesis.

Increasing ablation depth meant a deviation of the functional spot between the laser and sample surface from the laser focal point, resulting in the gradual reduction of ablation ability and efficiency. This finding is consistent with the results of Ji *et al*.^18^. More notable is that, for dentin, as ablation depth was increased, ablation efficiency quickly decreased and then gradually decreased - an effect that could be due to the creation of a smear layer formed during the mechanical cutting of the original dentin samples.

From the results of this study, we conclude that use of a reasonable laser fluence, a marginal degree of overlap between adjacent ablation lines and proper focusing of the laser on the ablation plane in real time are important for obtaining good ablation efficiency during the cavity preparation process of dental enamel and dentin using a femtosecond laser. Future studies are needed to characterize both the mechanism of femtosecond laser activity on enamel and dentin, as well as the quantitative mathematical model of three-dimensional ablation.

## Additional Information

**How to cite this article**: Chen, H. *et al*. Femtosecond laser for cavity preparation in enamel and dentin: ablation efficiency related factors. *Sci. Rep.* **6**, 20950; doi: 10.1038/srep20950 (2016).

## Figures and Tables

**Table 1:** Ablation depth under different laser fluences for enamel and dentin.

Fluence (J/cm^2^)	Number of Scanning Layers	Ablation depth, Mean ± SD (μm)	
		Enamel	Dentin
1.56	800	—	36.54 ± 1.23
3.13	800	55.01 ± 2.79	472.33 ± 16.37
4.69	400	209.31 ± 14.88	356.22 ± 8.65
6.25	200	143.71 ± 1.89	201.31 ± 7.63
7.81	200	159.85 ± 3.67	229.59 ± 5.54
